# Tough and Elastic α-Tricalcium Phosphate Cement Composites with Degradable PEG-Based Cross-Linker

**DOI:** 10.3390/ma12010053

**Published:** 2018-12-24

**Authors:** Michaela Rödel, Jörg Teßmar, Jürgen Groll, Uwe Gbureck

**Affiliations:** Department for Functional Materials in Medicine and Dentistry, University Hospital of Würzburg, Pleicherwall 2, 97070 Würzburg, Germany; michaela.roedel@fmz.uni-wuerzburg.de (M.R.); joerg.tessmar@fmz.uni-wuerzburg.de (J.T.); juergen.groll@fmz.uni-wuerzburg.de (J.G.)

**Keywords:** dual setting system, bending strength, calcium phosphate cement, composite material, HEMA, hydroxyapatite, free radical polymerization

## Abstract

Dual setting cements composed of an in situ forming hydrogel and a reactive mineral phase combine high compressive strength of the cement with sufficient ductility and bending strength of the polymeric network. Previous studies were focused on the modification with non-degradable hydrogels based on 2-hydroxyethyl methacrylate (HEMA). Here, we describe the synthesis of suitable triblock degradable poly(ethylene glycol)-poly(lactide) (PEG-PLLA) cross-linker to improve the resorption capacity of such composites. A study with four different formulations was established. As reference, pure hydroxyapatite (HA) cements and composites with 40 wt% HEMA in the liquid cement phase were produced. Furthermore, HEMA was modified with 10 wt% of PEG-PLLA cross-linker or a test series containing only 25% cross-linker was chosen for composites with a fully degradable polymeric phase. Hence, we developed suitable systems with increased elasticity and 5–6 times higher toughness values in comparison to pure inorganic cement matrix. Furthermore, conversion rate from α-tricalcium phosphate (α-TCP) to HA was still about 90% for all composite formulations, whereas crystal size decreased. Based on this material development and advancement for a dual setting system, we managed to overcome the drawback of brittleness for pure calcium phosphate cements.

## 1. Introduction

Bone is often described as natural miracle due to its impressive mechanical properties. On the one hand, it has a very high load-capacity of ~90–190 MPa [1] for cortical bone, while at the same time it provides elastic and ductile properties with a high fracture resistance. This high stability is based on its structural composition, as natural bone is a composite mainly consisting of apatite, collagen, and water [2,3]. This leads to the idea to mimic bone structure in synthetic materials by combining an inorganic phase based on calcium phosphate cements (CPCs) with an organic polymeric matrix.

CPCs are widely used as substitutes for non–load bearing defects. They are promising materials for replacement of damaged bone and formed from a solid raw powder and a liquid phase [4]. According to the chosen pH-values during setting reaction as well as different solubility products constants *K_sp_*, one can distinguish between brushite (DCPD, CaHPO_4_·2H_2_O; pH ≤ ~4.2) and hydroxyapatite (HA, Ca_5_(PO_4_)_3_OH; pH ≥ ~4.2) forming cements. The latter one is clinically a very well established system due to its outstanding properties like bioactivity, osteoconductivity [5] and stochiometric similarity to mineral components present in bone and teeth [6]. Furthermore, Bohner [7] describes an “excellent” biocompatibility of apatite CPCs despite their slow biodegradability. The drawback of such cements is their inherent brittleness and low tensile and bending strength, which can be overcome by either fiber reinforcement or by creating dual-setting cement systems [8]. The latter is characterized by a simultaneous cement setting reaction and polymerization of organic additives, which consequently form a homogenous and incorporated network. Previously described dual setting cement systems are mainly based on HA forming cements modified with organic matrices such as polyacrylamide [9], glycidyl methacrylated dextran [10] or six-armed star molecules functionalized with isocyanate groups as reactive termini (NCO-sP(EO-*stat*-PO)) [11]. Furthermore, Christel et al. [12] reported on a dual setting system with 2-hydroxyethyl methacrylate (HEMA) as water-soluble polymerizable monomer in the liquid solution. The gelation was performed via radical polymerization using ammonium persulfate (APS) and tetramethylethylenediamine (TEMED) as radical system. With incorporation of a poly-HEMA (pHEMA) network, mechanical properties could be improved without influencing workability and processability of this cement system. However, polymerization of this monofunctional monomer only results in non-cross-linked polymeric chains, which form a dense entangled and non-degradable network.

Here, we further modified this cement system by the addition of a degradable poly(ethylene glycol)-poly(lactide) (PEG-PLLA) cross-linker, which is thought to further ameliorate the mechanical properties of the system and increase bending strength and ductility [13]. Focusing on HA, which has a high similarity to the mineral component of bone tissue, the following study was performed using α-tricalcium phosphate (α-TCP) with a 2.5 wt%-Na_2_HPO_4_-solution as accelerator for the setting process. Both, PEG-PLLA hydrogels and cement composites were thoroughly characterized regarding their mechanical properties and the influence of the modification on the cement setting reaction was investigated. 

## 2. Materials and Methods

### 2.1. Cross-Linker Synthesis and Characterization

#### 2.1.1. Cross-Linker Synthesis

**Scheme 1:**
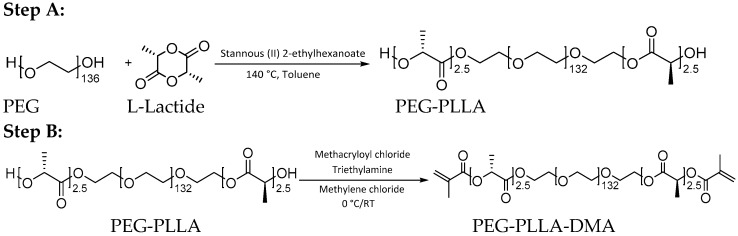
Reaction scheme of the two-step synthesis. Step **A** depicts the formation of poly(ethylene glycol)-poly(lactide) (PEG-PLLA), which was generated via ring-opening polymerization and tin-catalysis with stannous-based catalyst. In reaction **B**, terminal hydroxyl groups were functionalized with methacrylic acid groups (PEG-PLLA-DMA). RT = room temperature.

A linear triblock cross-linker composed of a PEG-backbone (Sigma Aldrich, Steinheim, Germany) and poly(lactid acid) (PLA)-spacers was synthesized via standard ring opening polymerization adapted from Sawhney et al. [14]. Briefly, beforehand PEG (Merck, Darmstadt, Germany) with a molar mass of 6 kDa and L-Lactide (Sigma Aldrich, used in a molar ratio of 1 to 5) were dried by azeotropic distillation with toluene (VWR, Ismaning, Germany) at 140 °C under oxygen-free conditions. Both solvent phases were combined in a round-bottom flask and stannous (II) 2-ethylhexanoate (Sigma Aldrich) was added as catalyst. The reaction mixture was stirred at 140 °C for 6 h and cooled to room temperature. Afterwards, toluene was evaporated twice with subsequent dissolution of the residual in dichloromethane (DCM, VWR), precipitation in cold diethyl ether (VWR) and drying under vacuum. The reaction is depicted in Scheme 1, step A. In a second step, the obtained PEG-PLLA with unmodified hydroxyl groups was dissolved in 200 mL dry DCM and cooled down to 0 °C in an ice bath under oxygen-free conditions. Triethylamine (Sigma Aldrich) and methacryloyl chloride (Sigma Aldrich) were added to the reaction mixture and stirred for 12 h at 0 °C and 36 h at room temperature. After filtration of the solvent, the hydrogel precursor was twice precipitated in cold diethyl ether. Finally, the product (PEG-PLLA-DMA) was dried under vacuum for several hours. Methacrylation of the intermediate PEG-PLLA is shown in Scheme 1, step B.

#### 2.1.2. ^1^H Nuclear Magnetic Resonance Spectroscopy

^1^H Nuclear Magnetic Resonance (NMR) spectra were recorded on a Bruker Fourier 300 at 300 MHz with deuterated chloroform (CDCl_3_; 98%; Euriso-top, Saint-Aubin Cedex, France) as solvent and referred to non-deuterated residual solvent peak as internal reference. For the measurement, 10–20 mg of polymer was weighed and dissolved in 0.5 mL of solvent.

#### 2.1.3. Gel Permeation Chromatography in *N,N*-Dimethylformamide

Gel permeation chromatography (GPC) measurements were performed using the analytical system Agilent Technologies 1260 Infinity (pump and refractive index detector) from Polymer Standards Service (PSS; Polymer Standards Service GmbH, Mainz, Germany). *N,N*-Dimethylformamide (DMF; Carl-Roth, Karlsruhe, Germany) was added with 1 g·L^−1^ lithium bromide (LiBr; Sigma Aldrich) and used as eluent and solvent of the samples. A flow rate of 1.0 mL·min^−1^ was chosen to perform single GPC runs. Column system of the device was composed of one precolumn (PSS GRAM) and two columns with a length of 300 mm, a width of 8 mm and a particle size of 10 µm (PSS GRAM). For calibration, PEG standards (PSS) were used.

#### 2.1.4. Matrix Assisted Laser Desorption-Ionization Time-of-Flight Mass Spectrometry

The linear co-polymer was characterized regarding molar mass by matrix-assisted laser desorption-ionization time-of-flight mass spectrometry (MALDI-ToF MS). All MALDI mass spectra were acquired on a time of flight mass spectrometer (Bruker Daltonics autoflex II, Bruker Daltonik GmbH, Leipzig, Germany), equipped with a SCOUTTM MTP MALDI Ion Source. The following procedure was adapted from Marie et al. [15]. Standards were prepared by mixing a solution of 10% cesium triiodide (CsI_3_, Sigma Aldrich) in acetonitrile (ACN, Sigma Aldrich) with equimolar volumes (1 µL) of a 2.5% solution of *trans*-2-[3-(4-*tert*-Butylphenyl)-2-methyl-2-propenylidene]malononitrile (DCTB, Sigma Aldrich) in ACN. Subsequently, the mixture was spotted (V = 1 µL) on the aluminum maldi target (Bruker Daltonics MTP 384 massive target T; Part No.: 26755). Samples were then prepared by mixing the polymer solution (10 mg·mL^−1^ in CHCl_3_) with the matrix solution (50 mg·mL^−1^ DCTB in CHCl_3_). A volume of 1 µl was spotted on the maldi target. 100 shots at 20 Hz of a 337 nm nitrogen laser (LTB MNL205 BA352A61) were applied and spectra were accumulated. 

#### 2.1.5. Fourier Transform Infrared-Spectroscopy

Fourier transform infrared (FTIR) spectra were recorded by a Nicolet™ iS™10 Spectrometer (Thermo Fisher Scientific, Waltham, MA, USA) within a wave number range of 4000–650 cm^−1^ via attenuated total reflexion (ATR) mode. Therefore, scan number was selected with 16 scans per measurement and a resolution of 4.

### 2.2. Pure Hydrogel Production and Characterization

#### 2.2.1. Determination of Gelation Point via Rheological Analysis

The gel point of each systems was monitored by oscillatory time sweep with the rheometer MCR301 (Anton Paar GmbH, Graz, Austria). A plate-plate-geometry with a diameter of 25 mm was used for the measurements at a selected temperature of 16 °C. Time sweeps were carried out with a constant amplitude *γ* of 1% and a constant frequency *f* of 1 Hz. Storage (*G’*) and loss modulus (*G’’*) were measured during a time frame of 30 min. The solutions for hydrogel formation were prepared according to Table 1. Therefore, a volume of 1 mL for each solution was used for rheological characterization. Abbreviations for the different batches are as follows: 40 wt% HEMA (H40), 10 wt% PEG-PLLA-DMA (P10), and a combination of both (H40P10). According to the very fast gelation kinetic of pure hydrogels composed of the PEG-PLLA–based cross-linker, the concentration was reduced to 10 wt%. However, a clear trend and prediction for higher concentrations like 25 wt% can be drawn. 

#### 2.2.2. Degradation and Swelling Behavior

For investigations concerning swelling behavior of the different hydrogel batches, solutions were prepared analogously to composition overview in Table 1. Specimens were gelled in round silicone molds with a diameter of 10 mm and a height of 3 mm and put in a drying chamber with 37 °C for 5 min. In order to characterize the hydrogel systems, these small discs were weighed over a period of approximately 10 weeks and put into glass vials. Afterwards, they were placed in 5 mL of phosphate buffered saline (PBS) (pH 7.4) and stored on a shaking gadget at 37 °C in an oven. The equilibrium water content (*EWC*) as weight percentage was calculated using Equation (1). *W_s_* and *W_d_* are the weights of swollen and dry specimens, respectively.

(1) $$EWC=\frac{W_{s}-W_{d}}{W_{s}}\times 100\%$$

Furthermore, the volumetric swelling ratio *Q_ν_* as another expression for swelling degree was determined by the quotient of swollen polymer (*W_s_*) and dry polymer (*W_d_*). Both parameters were determined for two different time points: immediately after production and after 4 h.

(2) $$Q_{v}=\frac{W_{s}}{W_{d}}$$

#### 2.2.3. Degree of Cross-Linking for Synthesized Hydrogel Precursor via Determination of Methacrylic Acid

**Scheme 2:**
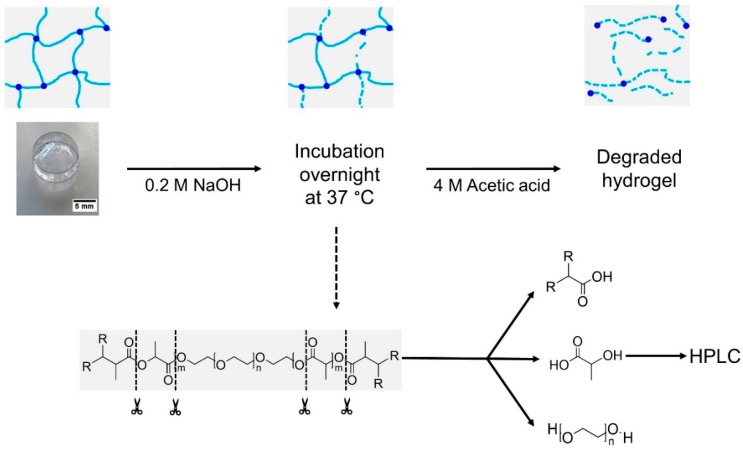
Schematic overview of determined degree of methacrylation as well as amount of cross-linked free methacrylic acid. Different steps of the procedure starting from basic hydrolysis with incubation overnight to protonation of methacrylic acid until a complete degradation of the hydrogel is obtained. This compound was analyzed via HPLC according to a procedure of Abbadessa et al. [16].

Degree of cross-linking as well as methacrylation for PEG-PLLA-DMA-based hydrogels were both investigated using a method adapted from Abbadessa et al. [16]. Round specimens were produced according to the procedure described in 2.1 (composition see Table 1). Ester bonds of the polymeric network were basically hydrolyzed by addition of 0.2 M NaOH-solution (4 mL; produced out of NaOH, Merck). Therefore, samples were incubated at 37 °C overnight. For protonation of released methacrylic acid, 3 mL of 4 M acetic acid (produced out of acetic acid, glacial, Alfa Aesar, Kandel, Germany) were pipetted to the gels. This process is illustrated in Scheme 2. 

After degradation verified by transparent solutions, methacrylic acid was detected via high-performance liquid chromatography (HPLC; Shimadzu (LC-20AT) with autosampler (SIL-20AC), Kyoto, Japan) equipped with a C18 column (Luna 5 µm C18(2) 100 Å, LC Column 150 mm × 4.6 mm) and a photodiode array detector (SPD-M20A) with a chosen wave length *λ* of 210 nm. Eluent composed of water/ACN (Sigma Aldrich) in a ratio of 85:15 with a pH of 2 (adjusted with perchloric acid, Merck) and a set flow of 1 mL·min^−1^. Calibration was performed using solutions of methacrylic acid in the same eluent within a concentration range of 0.55–279.47 µg·mL^−1^. For standards as well as samples, injection volume was set to 10 µL.

Calculation of cross-linked methacrylic acid functionalities as measure for integrity in network formation was performed according to Equation (3).

(3) $$\%\mathrm{of}\;\mathrm{cross-linked}\;\mathrm{methacrylic}\;\mathrm{acid}=100\%-\frac{\%\mathrm{of}\;\mathrm{methacrylic}\;\mathrm{acid}\;\mathrm{in}\;\mathrm{gels}}{\mathrm{total}\;\mathrm{of}\;\mathrm{methacrylic}\;\mathrm{acid}\;\mathrm{in}\;\mathrm{contol}}$$

### 2.3. Cement Composite Formation and Characterization

#### 2.3.1. Synthesis of α-Tricalcium Phosphate

α-Tricalcium phosphate (α-TCP, Ca_3_(PO_4_)_2_) was synthesized by sintering a mixture of monetite (CaHPO_4_, 2.15 mol; J.T.Baker, Fisher Scientific GmbH, Schwerte, Germany) and calcium carbonate (CaCO_3_, 1 mol; Merck) at 1400 °C for 5 h followed by quenching to room temperature and grinding in a ball mill (Retsch, Haan, Germany) for 4 h.

#### 2.3.2. Production of Inorganic Reference and Dual Set Composites

**Scheme 3:**
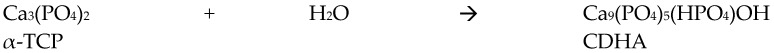
Cement setting reaction to calcium deficient hydroxyapatite (CDHA) with α-tricalcium phosphate (α-TCP) as raw powder reactant in combination with 2.5% disodium phosphate solution.

As inorganic reference, pure HA samples with a powder-to-liquid ratio (PLR) of 3 g·mL^−1^ were produced by mixing 2.5% disodium phosphate solution (Na_2_HPO_4_; Merck) with α-TCP raw powder. The obtained setting reaction sets to a matrix of calcium deficient hydroxyapatite (Ca_9_(PO_4_)_5_HPO_4_OH; CDHA) within several days. This process is shown in Scheme 3.

For dual setting composites, the radical starter ammonium persulfate (APS; 0.5 wt%; Sigma Aldrich) was homogeneously mixed with α-TCP raw powder (99.5 wt%) using an electric coffee grinder for 60 s. Base of the liquid phase was a 2.5% Na_2_HPO_4_-solution (Merck) with addition of the radical catalyst *N,N,N’,N’*-tetramethylethylenediamine (TEMED; 2.5 wt%, Sigma Aldrich). Three different formulations were tested: (i) the first batch contained 40 wt% hydroxyethyl methacrylate (HEMA; H40; Sigma Aldrich) added to the liquid phase. A (ii) second formulation combined the short-chain monomer HEMA (40 wt%) with the synthesized polymeric cross-linker PEG-PLLA-DMA (10 wt%; H40P10). Third mixture (iii) was based on the pure synthesized PEG-hydrogel precursor (25 wt%; P25). The selected PLR was 3 g·ml^−1^ for all three formulations. Cement setting to HA as well as radical polymerization was initiated by homogenous mixing of cement powder and liquid on a glass slab.

#### 2.3.3. Initial Setting Time Determination via Gillmore Needle Test

Initial setting time was determined via Gillmore needle test in a chamber with a relative humidity of 95–100% and a temperature of 37 °C. Samples were prepared with a capacity of 0.8 g and a PLR of 3 g·mL^−1^. Time measurement was started after addition of the reaction solution to the powder phase. In case of no mark of the plunger could be seen on the set cement, time was stopped.

#### 2.3.4. Phase Composition and Conversion via X-ray Diffraction and Rietveld Refinement

X-ray diffraction (XRD) patterns of samples were determined using the D5005 diffractometer (Siemens, Munich, Germany) with monochromatic Cu K_α_ radiation. Data measurements were performed in the range of 2*θ* = 20–40° with a step size of 0.02° and a normalized count time of 1.5 s·step^−1^. Phase composition was checked by means of Joint Committee on Powder Diffraction Standards (JCPDS) reference patterns for hydroxyapatite (PDF Ref. 09-0432), α-TCP orthorhombic (PDF Ref. 09-0348) and α-TCP monoclinic (PDF Ref. 09-0359). Rietveld refinement was accomplished with the software TOPAS 2. For calculation of the phase composition with the amount of α-TCP and HA, respectively, the used raw file data named “α-TCP 1h milled, 24 h hardened” was used with respect to ICDD data base and the reference numbers of HA (090432) and α-TCP (290359). HA crystal size was determined via the characteristic signal at *θ* = 26°. 

#### 2.3.5. Surface Analysis via Scanning Electron Microscopy

Scanning electron microscopy (SEM) images were recorded with the scanning electron microscope CB 340 from Zeiss (Oberkochen, Germany) at an acceleration voltage of 2.0 kV. For a better electrical conductivity, surfaces of the samples were sputtered with a 4 nm thick platinum layer using the High Vacuum Coater Leica EM ACE 600 (Emitech, Molfetta, Italy).

#### 2.3.6. Mechanical Characterization—Four-Point Bending and Compression Test

For mechanical characterization, both (i) four-point bending test and (ii) compressive strength test were performed using the static mechanical testing machine Zwick/Roell Z010 (Zwick/Roell, Ulm, Germany). For testing of bending strength, a 2.5 kN load cell was used with a crosshead speed of 1 mm·min^−1^. Samples were prepared in cuboid silicon rubber molds with a dimension of 3 × 4 × 45 mm^3^. Additional parameters were determined and calculated, respectively: toughness as area under load-displacement curves, elasticity as function of deformation according to Equation (4), and E-modulus as function of 4-point bending test according to Equation (5).

(4) $$\mathrm{Elasticity}/\%=\frac{4\times a\times dL}{lb^{2}}$$

(5) $$E-\mathrm{modulus}/\mathrm{Gpa}=\frac{3\times \left(l_{a}\times {\left(l_{b}\right)}^{2}\times \left(X_{l}-X_{h}\right)\right)}{4\times dc\times b\times a^{3}}$$

Compressive strength testing was carried out by using a 10 kN load cell with a crosshead speed of 1 mm·min^−1^. For this test, pastes were prepared in silicon rubber molds with a dimension of 6 × 6 × 12 mm^3^. 

During different time points of testing, samples were stored in PBS buffer over eight days with buffer change and analysis of pH-level every second day. The used chemicals potassium chloride (KCl; Merck; 0.2 g per 1000 mL), sodium chloride (NaCl; Sigma Aldrich; 8.0 g per 1000 mL), disodium hydrogenphosphate (Na_2_HPO_4_; Merck; 1.11 g per 1000 mL), and potassium dihydrogenphosphate (KH_2_PO_4_; Merck; 0.2 g per 1000 mL) were dissolved in a volumetric flask, and pH-level (InoLab Level 2 WTW, Weilheim, Germany) was adjusted with 0.1 M sodium hydroxide solution (NaOH; Merck) to 7.4.

#### 2.3.7. Statistical Analysis

Significant differences* (*(*p* < 0.05) and **(*p* < 0.001) between two groups) were tested with SigmaPlot 12.5 (Systat Software, Erkrath, Germany) performing ANOVA and post-hoc Tukey test. 

## 3. Results

### 3.1. Cross-Linker and Hydrogel Characterization

#### 3.1.1. Cross-Linker Characterization

**Figure 1:**
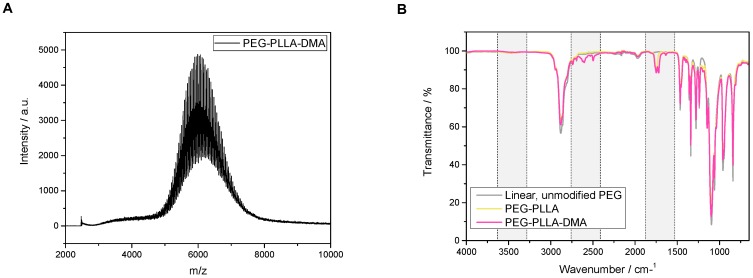
MALDI-ToF analysis (**A**) and FTIR spectra (**B**) of the synthesized PEG-PLLA-DMA cross-linker. PEG-PLLA-DMA: dimethacrylated poly(ethylene glycol)-poly(lactide).

The successful synthesis of the PEG-PLLA cross-linker was previously proved by ^1^H-NMR spectroscopy [13]). Further DMF-GPC-analysis in the current study confirmed a monomodal distribution and narrow dispersity of Ð = 1.08 (M_n_ = 6789 g·mol^−1^, M_w_ = 7330 g·mol^−1^ in comparison to the expected value of 6498 g·mol^−1^; chromatogram not depicted in this publication). Also, MALDI-ToF measurements showed a mean molar mass of 6077 Da and clearly detectable PEG-units with *m*/*z* = 44 (see Figure 1A). Moreover, FTIR measurements revealed additional absorption bands for the first and second ester bond (*ν* = 2300 cm^−1^ and *ν* = 1725 cm^−1^). Unmodified, linear PEG with free hydroxy groups at both sides showed a strong signal of the PEG-backbone with its CH-valence vibration (*ν* = 2875 cm^−1^) and a slight alcohol OH-stretch at *ν* = 3455 cm^−1^. With esterification of the end functionalities, another signal appears in the wavenumber range of the corresponding carboxylic ester stretch at *ν* = ~1725 cm^−1^. In the second step of the synthesis, terminal PLA-units are transformed with methacrylic acid units and the incorporation of double bonds. The equivalent alkenyl C=C-double bond stretch appears at *ν* = ~2300 cm^−1^ (see Figure 1B). 

#### 3.1.2. Hydrogel Characterization

**Figure 2:**
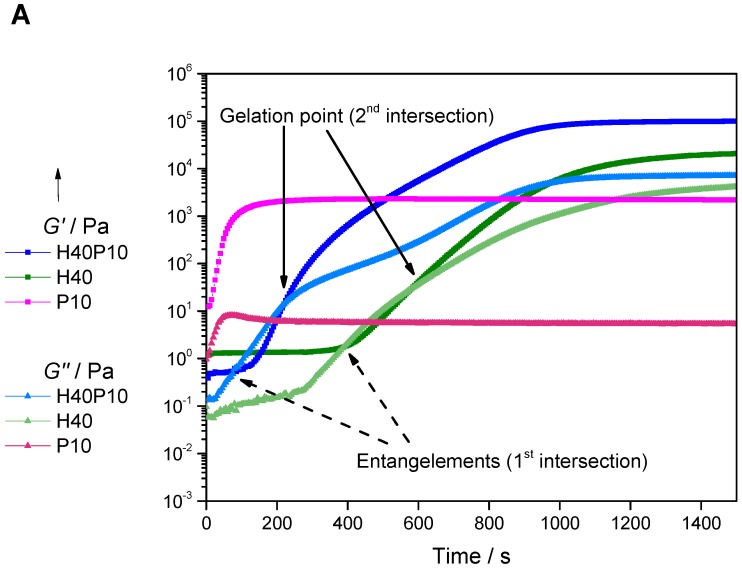

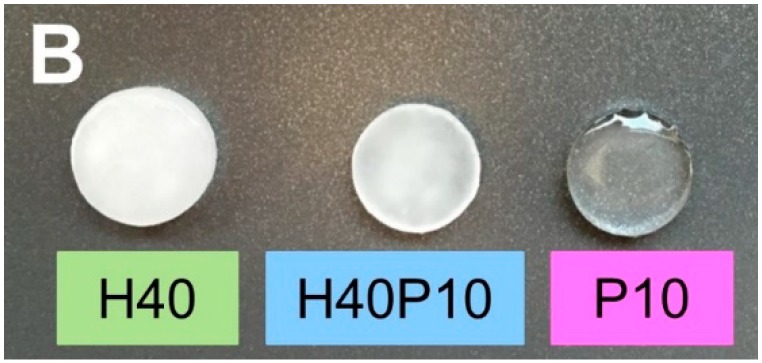
Gelation point measurements (**A**) of the different pure hydrogel systems composed of 40 wt% 2-hydroxyethyl methacrylate (HEMA) (H40), 10 wt% PEG-PLLA-based hydrogel precursor (P10) or a combination of both (H40P10) at 16 °C over 30 min. Image (**B**) depicts the structure of the different hydrogel samples with a very turbid and milky look of the HEMA sample to the clear and transparent PEG-PLLA-hydrogel.

In order to characterize the gelation process, which occurs simultaneously to cement setting reaction in the dual component system, the gel point was determined via oscillating rheology. Therefore, time sweeps of the three different polymeric systems containing 40 wt% HEMA, 10 wt% PEG-PLLA-based hydrogel precursor (P10), or a combination of both (H40P10) were investigated (see Figure 2A). Two cross-over points for the formulations with HEMA could be observed. The first was detected after 385 s (= 6 min 25 s), with a second intersection after 585 s (= 9 min 45 s). Due to the effect of short chain entanglements or aggregation of the monomers, there is a domination of the elastic properties at the beginning of the measurement. After destruction of the formed pre-structures in solution, the loss modulus *G’* dominates and the starting properties for an initial gelation are regained. The second intersection of storage modulus *G’’* and loss modulus *G’* is determined as gel point of the polymeric systems. For the hydrogel system composed of HEMA and cross-linker (H40P10), the first intersection is reached earlier after 70 s (= 1 min 10 s) due to an increase in reaction kinetics with fast gelation of the cross-linker. With respect to this higher reactivity of the synthesized cross-linker, it was not possible to measure the intersection of both moduli for the hydrogel formation of the batch based on 10 wt% PEG-PLLA-DMA (P10) in the given time frame of less than one minute caused by initializing and starting processes of the rheometer. 

**Figure 3:**
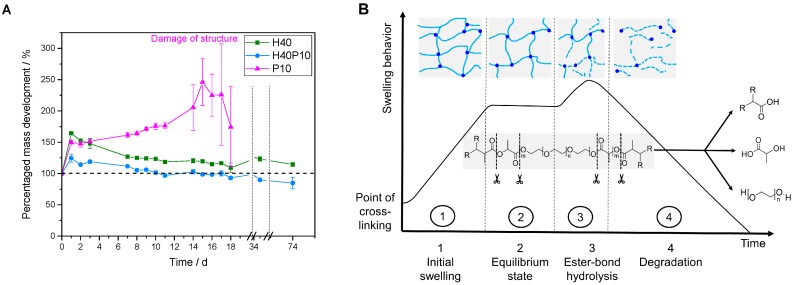

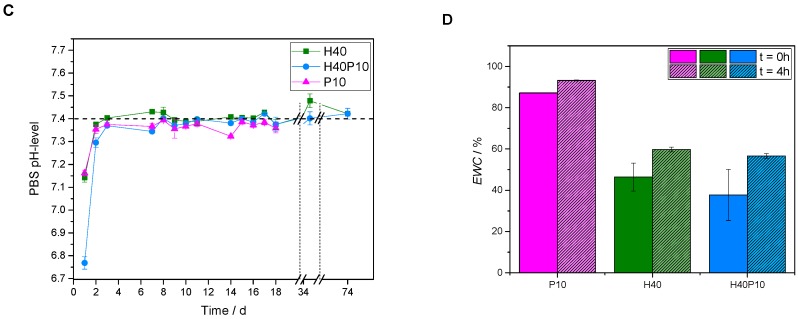
Overview of the gel characterization for the specimens with 40 wt% HEMA (H40), a combination of HEMA and cross-linker (H40P10) and the pure PEG-PLLA_DMA triblock hydrogel with 10 wt% polymer (P10). The different batches were analyzed regarding mass development in PBS buffer at pH 7.4 (**A**), pH-level of the storage medium (**C**) as well as *EWC* (**D**). Additionally, a schematic curve of the degradation study corresponds to obtained data (**B**).

With addition of the cross-linker, the elastic behavior of the system could be improved in the way that the elastic modulus, which refers to the stored deformation energy in the system, reached a level of about 10^5^ Pa. The elasticity corresponds also to the water content in the different gel systems. The pure PEG-PLLA-gel system behaved like a typical hydrogel system with a wide polymer network, exhibiting the highest *EWC* (see Figure 3D) and a duplication of *Q_ν_* from 7.81 ± 0.05 for t = 0 h to 14.82 ± 0.40 after 4 h. Both batches containing HEMA (H40 and H40P10) were dominated by the presence of a high number of short chain molecules and a denser hydrogel network. Consequently, produced samples were not transparent and showed lower *Q_ν_*-levels (H40: t(0 h) = 1.90 ± 0.26, t(4 h) = 2.49 ± 0.07; H40P10: t(0 h) = 1.66 ± 0.29, t(4 h) = 2.31 ± 0.06). Gels of the different batches are depicted in Figure 2B.

#### 3.1.3. Degradation Behavior of Hydrogels in PBS

Regarding the degradation behavior of the different samples, the samples containing pHEMA in a relatively high amount of 40 wt% (H40) showed an initial mass increase with a slight decrease to constant values over 10 weeks. Furthermore, no swelling was observed and the constructs were still round shaped and without any cracks or missing parts. This effect of a pHEMA-based polymeric network was also investigated for gels combined with high molecular weight cross-linker. By incorporation of a degradable hydrogel precursor in a weight amount of 10 wt% (H40P10), the short chain-polymeric system was still dominating. After one day, mass increased about 25% but turned into a stable hydrogel formation over the time with an almost constant mass for approximately 10 weeks observation (see Figure 3A). 

Additionally, hydrogels of pure PEG-PLLA-DMA-cross-linker (P10) were analyzed. At the beginning, an increase in mass of about 50% was observed. Hydrogels behaved like expected for ester-containing substances (see Figure 3B): shortly after the cross-linking, an equilibrium state for the water-up-take of the hydrogel network was reached. This period lasted for about 12 days, until significant ester bond hydrolysis started. Samples became instable, fragmented and were broken after 18 days (see Figure 3A; Damage of structure). Due to the breakdown of the structure indicated by the decrease in mass and high standard deviations due to the loss of fragments, a mass determination was feasible any more. This was only observed for P10, whereas that for HEMA-containing formulations the short and low molecular weight polymers are the dominating factor. In addition to mass change development, pH-values of PBS storage solution were measured. First pH-levels of all batches (day 1) started in acidic regions of 6.75 for H40P10 and 7.15 for H40P10 as well as P10. After three days, reached values were in the physiological range about a pH of 7.4 indicating very low release of acidic degradation products (see Figure 3C). To characterize also uptake of water, *EWC* of the different hydrogels was determined in the range of immediately after production (t = 0 h) and after 4 h. Hydrogels based on the self-synthesized cross-linker showed a very high *EWC* of about 80–90% after 4 h. In contrast to that, gels based on pHEMA (H40 and H40P10) reached lower *EWC*-values of about 40 % for the first time point and increased up to 50% after 4 h (see Figure 3D). 

#### 3.1.4. Determination of Methacrylate Units Involved in Gelation for PEG-PLLA-DMA–Hydrogels

**Figure 4:**
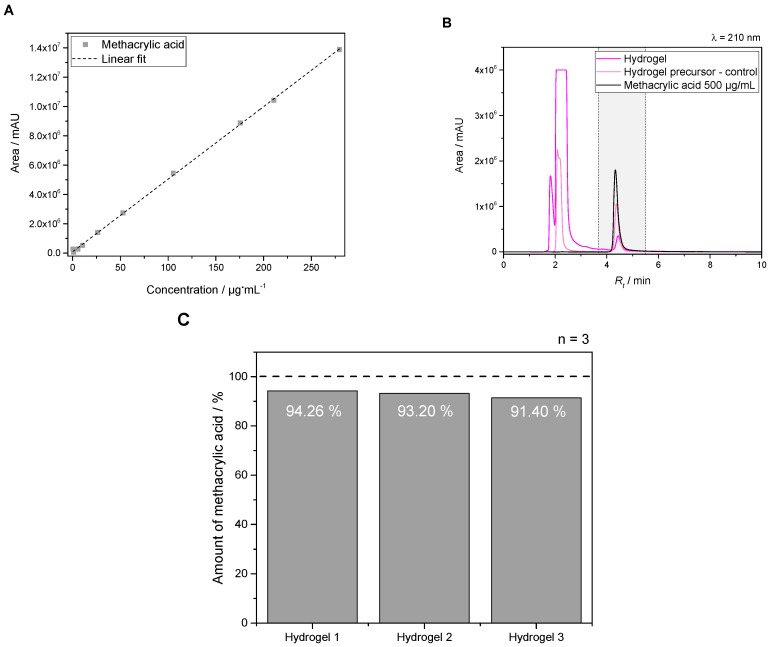
Determination of methacrylic acid units for degree of modification as well as incorporation of functions for gelation. High-performance liquid chromatography (HPLC) was used as method for quantification. Calibration curve of methacrylic acid standards (**A**) as well as a chromatogram (**B**) showing hydrogel, control and standard profile. Furthermore, the total amount per PEG-PLLA–based hydrogel was in the range of 92.95% ± 1.45 (**C**).

Free methacrylic acid added to the eluent was used as standards for calibration within the range of 0.05–280 µg·mL^−1^. A linear correlation with a regression coefficient R^2^ of 0.9997 and a linear equation of y = 49,645x + 63,906 was obtained for calibration (see Figure 4A). Furthermore, retention time (R_t_) of the analyzed compound was 4.3 min within a run duration of 10 min. Figure 4B gives an overview of a chromatogram at *λ* of 210 nm showing the pure hydrogel precursor as control, the hydrolyzed hydrogel itself as well as a methacrylic acid standard with a concentration of 0.5 mg·mL^−1^. HPLC measurements verified a very high degree of cross-linking efficiency by reaching 92.95% ± 1.45 of acid units for PEG-PLLA-DMA-hydrogels (see Figure 4C).

### 3.2. Inorganic Reference and Composite Characterization

#### 3.2.1. Determination of Initial Setting Time

**Figure 5:**
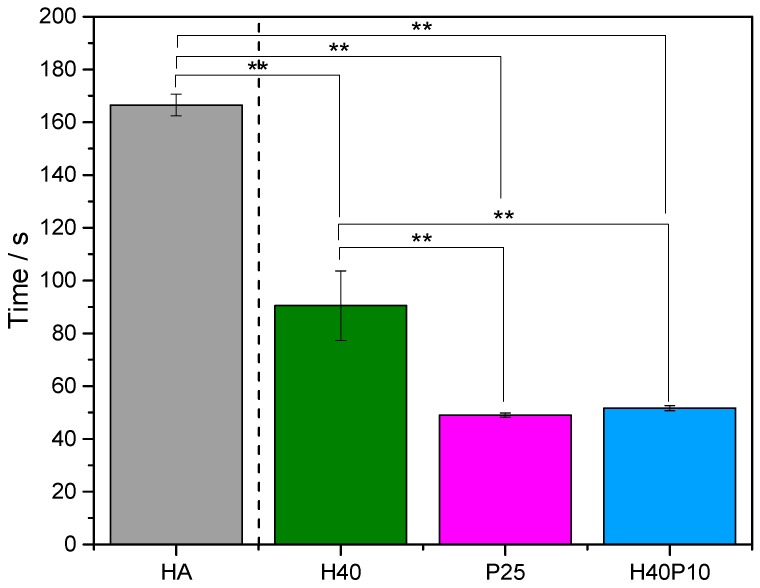
Initial setting times of pure inorganic reference HA in comparison to the composite formulations with 40 wt% HEMA (H40), HEMA in the same concentration combined with 10 wt% degradable cross-linker (H40P10) as well as a hydrogel phase produced out of 25 wt% polymer (P25). ** *p* < 0.001.

Initial setting times of pure HA and all composite formulations were analyzed via Gillmore needle test. Significant differences in setting times were observed with about 180 s (= 3 min) for the reaction of α-TCP to HA without any additives in the liquid or solid phase, respectively. Simultaneous gelation reactions that run in parallel to cement setting decreased the initial setting time to less than 120 s (= 2 min) for a pHEMA-based composite material (H40). Batches with 25 wt% polymeric hydrogel precursor showed even faster setting reactions less than 60 s (= 1 min). This polymer was the dominating factor for the as well very quick conversion and concurrent hydrogel formation for batch H40P10 with 40 wt% HEMA and 10 wt% cross-linker (see Figure 5).

#### 3.2.2. Analysis of Parameters for Biological and Material Technological Relevance

**Figure 6:**
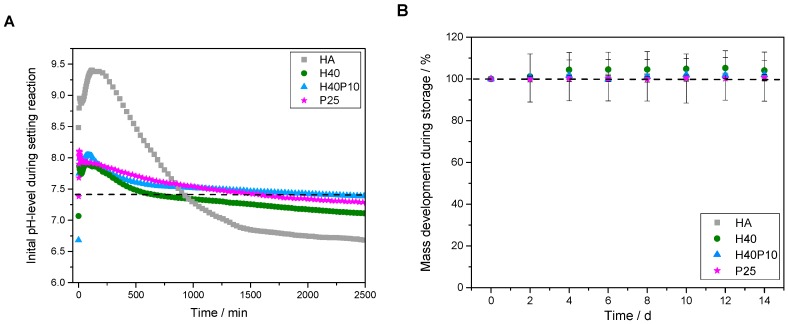

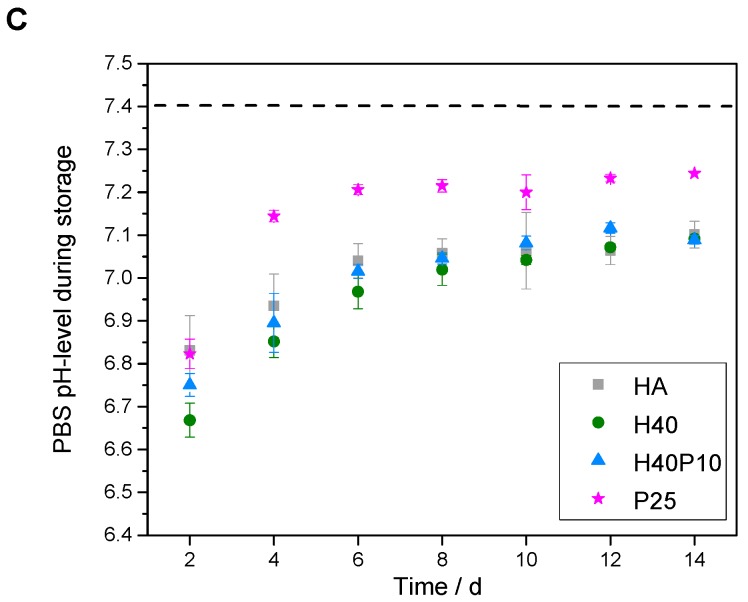
Analysis of different parameters for characterization of composite formulations. Regarding material technological relevance, initial pH-level during setting was measured (**A**). Furthermore, mass development during storing was observed (**B**). With respect to biological relevance, pH-level of PBS storage solution was determined (**C**).

The additional polymer phases incorporated in inorganic matrix also influenced initial pH-development. Pure HA showed a setting reaction under alkaline conditions with a basic pH-maximum of 9.4 after 2 h. A drop down of the pH-level after 24 h resulted in a still decreasing plateau phase under the physiological pH-level (see dotted line in Figure 6A). By addition of pHEMA and/or PEG-PLLA-based cross-linker, respectively, maximum pH-values decreased to a pH of 8 with a relatively constant and slow shift to 7.4. No swelling or increase in mass was observed over 8 d of storage (see Figure 6B). Moreover, all PBS media showed acidic conditions after 2 d in the range of 6.7–6.9. Over two weeks, pH-values increased up to 7.0–7.2 and approximated a pH-level of 7.4 (see Figure 6C).

#### 3.2.3. XRD Measurements and Rietveld Refinement

**Figure 7:**
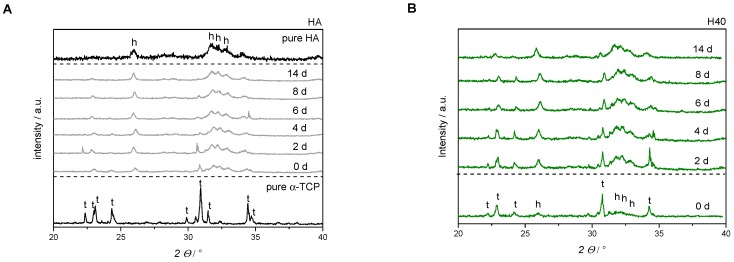

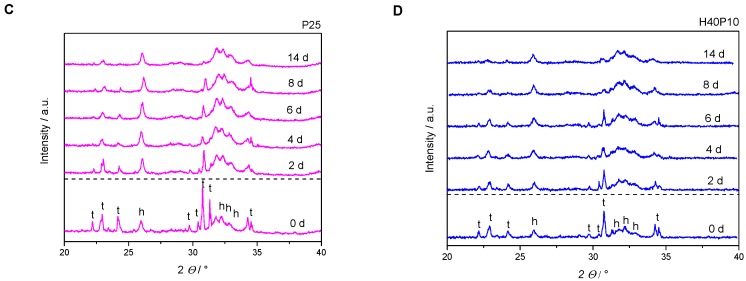
XRD-diffractograms of pure hydroxyapatite (HA) in comparison with composite batches. All formulations converted to HA over 14 days. With increasing polymer content, amorphous phases increased. Furthermore, a higher polymeric content also increased the time frame for HA formation (h = diffraction pattern HA, t = diffraction pattern α-TCP). (**A**) Inorganic reference HA in comparison to the reference diffraction pattern of pure α-TCP and HA, respectively; (**B**) HA with polymeric addition of 40 wt% HEMA (H40) in liquid phase; (**C**) HA with polymeric addition of 25 wt% synthesized hydrogel precursor (P25) in liquid phase; (**D**) HA with combination of 40 wt% HEMA and 10 wt% cross-linker (H40P10) in liquid phase.

Particles or a second phase in a CPC matrix always influence conversion rate as well as crystal growth. Their characterization was performed for HA as pure inorganic reference as well as composite materials. Without polymeric addition, XRD-diffractograms showed a decrease in α-TPC signal intensity and corresponding increase for the prominent HA diffraction pattern at 26°. This disappearance revealed an almost complete conversion after 14 days of storage under physiological conditions (100% relative humidity, 37 °C). Therefore, both reference pattern of the reactant (α-TCP) and the product (HA) were added (see Figure 7A). In general, a hydrogel incorporation based on pHEMA and/or cross-linker resulted in a slower conversion and setting reaction from α-TCP to HA (see Figure 7B–D). Moreover, with added polymeric phase, diffraction patterns were slightly shifted from crystalline to a more amorphous character for the composite materials. 

**Figure 8:**
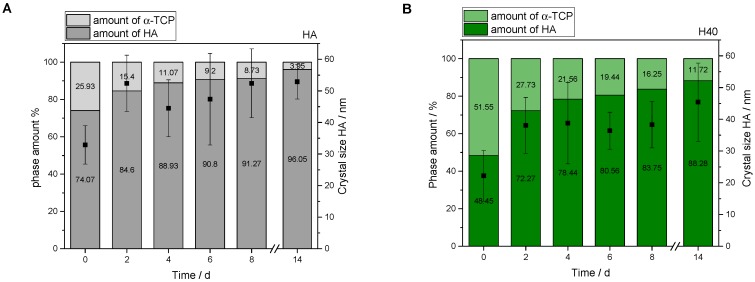

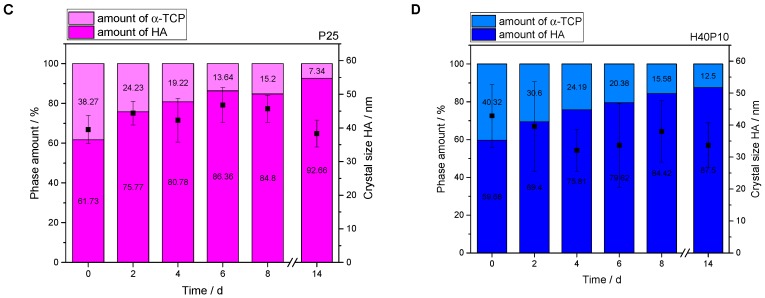
Overview of Rietveld refinement with analysis of HA crystal size. (**A**) Inorganic reference HA; (**B**) HA with polymeric addition of 40 wt% HEMA (H40) in liquid phase; (**C**) HA with polymeric addition of 25 wt% synthesized hydrogel precursor (P25) in liquid phase; (**D**) HA with combination of 40 wt% HEMA and 10 wt% cross-linker (H40P10) in liquid phase.

As mentioned above, with addition of a second hydrogel phase, transformation to HA was not as fast and complete as for the pure reference, where a hardening rate of nearly 96% was reached after 14 days (see Figure 8A). The combination of HEMA with 10 wt% cross-linker (H40P10) had the biggest influence on the transformation, given that after storage in PBS-buffer, the conversion rate was about 10% lower. The conversion rate decreased and HA crystal size was reduced by about 20 nm (see Figure 8D). Samples containing only pHEMA as hydrogel system (H40), especially at the starting point, showed a distribution of half raw material and final setting product (see Figure 8B). However, this imbalance with respect to the desired CPC formation recovered quickly after two days and led to 88% HA amount at the end with 12% residual α-TCP, respectively. Regarding batch P25 with gelled PEG-PLLA-DMA-precursor, size of HA crystals was about 38 nm and thus smaller in comparison to pure inorganic matrix. This polymeric system had less influence on the conversion and resulted in almost 92% of the desired mineral structure (see Figure 8C). 

#### 3.2.4. Microscopical Surface Characterization

**Figure 9:**
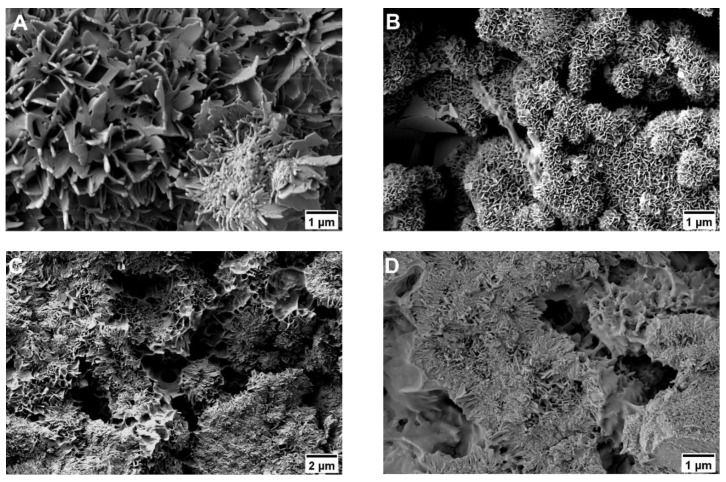
Scanning electron microscopy (SEM) images with different magnifications and details of the samples for surface characterization of HA and the different composite formulations H40, P25 and H40P10 after 8 d of storage. (**A**) Pure inorganic cement matrix showing typical HA needle-like structures (HA); (**B**) Batch with 40 wt% HEMA-addition depicting both, a successful setting and HA-formation as well as gelation (H40); (**C**) Smooth areas covering HA-needles and platelet structures indicating hydrogel formation for the batch containing 25 wt% hydrogel precursor PEG-PLLA-DMA (P25); (**D**) Combination of HEMA (40 wt%) and cross-linker (H40P10) resulted in a successful conversion to HA with an incorporated hydrogel mix of pHEMA and cross-linked PEG-PLLA-based polymers.

Results of Rietveld refinement regarding HA crystal size were also confirmed by SEM images. At different time points, samples were analyzed with respect to organic structures, their appearance as well as distribution in main parts of the cracking surfaces. HA references verified the typical plate-like and rosette-oriented growth of crystals (see Figure 9A). The same form and shape, but smaller needles, were observed for the composite materials H40, P25, and H40P10. Polymeric films in the samples that were dried during the transfer in the microscope covered inorganic structures. With higher magnifications, the influence of the hydrogel phase on HA crystal structure was clearly visible: needles were smaller and not as defined with clear demarcations at crystal borders (see Figure 9B–D). 

#### 3.2.5. Bending Strength

**Figure 10:**
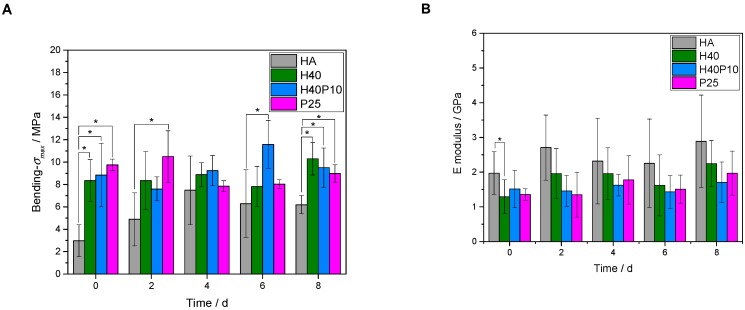

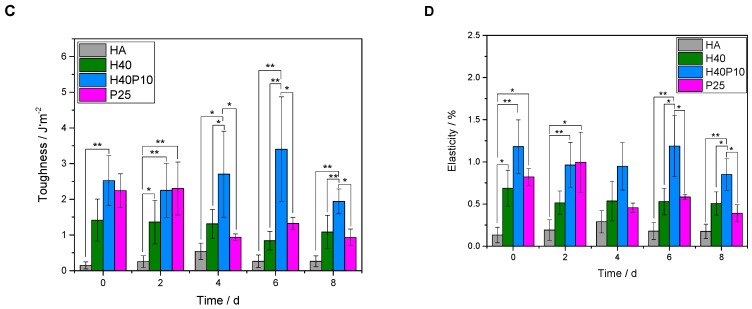
Overview of different parameters calculated out of four-point bending test. First, maximum bending strength of all batches over a period of eight days was determined (**A**). Furthermore, a calculation of relevant measures for evaluating influence of polymeric additions was performed: E-modulus (**B**), toughness (**C**), and elasticity (**D**) of the system. * *p* < 0.05; ** *p* < 0.001.

An increase in bending strength, as well as ductility and flexibility, was achieved by addition of the polymeric phase. Four-point bending strength could be significantly increased from about 6 MPa for the pure reference without polymeric additions to about 9 MPa for the samples with organic hydrogel phase (see Figure 10A). For different polymers, highest value of about 10 MPa was observed for the combination of 40 wt% HEMA with 10 wt% cross-linker (H40P10). Although pure hydrogel phases based on pHEMA seemed to have higher viscous and elastic behavior (*G’* respectively *G’’* with highest value, see Figure 2A), a four-point-bending strength in the same range of batch H40 could be measured for the pure cross-linker, i.e., the hydrogel system based on degradable methacrylate modified triblock PEG-PLLA-DMA-monomers. Over the time frame of 8 days, all dual-setting systems showed a relatively stable value of four-point bending strength in comparison to the reference where the strength increased related to the time-dependent hardening process of the cement. HA references had higher E-modulus values between 2 and 3 GPa in comparison to the dual set formulations. Lowest E-moduli and thus highest elasticity and lowest slope were determined for samples containing 25 wt% cross-linker (P25). This observation refers to the determined density of hydrogels and *EWC* showing highest elastic behavior (see Figure 10B). Highest energy absorption capacity and toughness was given by the combination of both, short and long chain polymers that formed an incorporated, homogenous hydrogel network (H40P10, see Figure 10C). Calculated elasticity was significantly advanced by the different hydrogel phases whereas a combination of 40 wt% HEMA and 10 wt% PEG-PLLA-based hydrogel precursor showed the overall highest effect (see Figure 10D). 

#### 3.2.6. Compressive Strength

**Figure 11:**
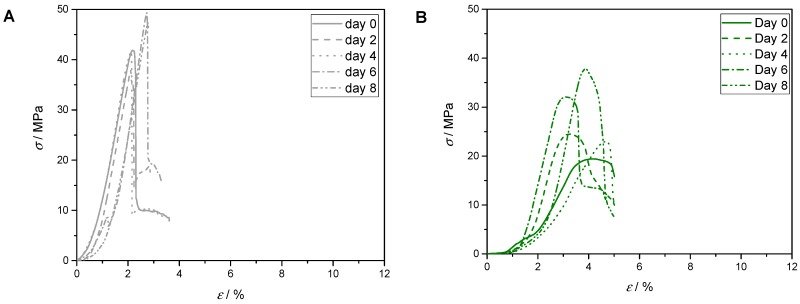

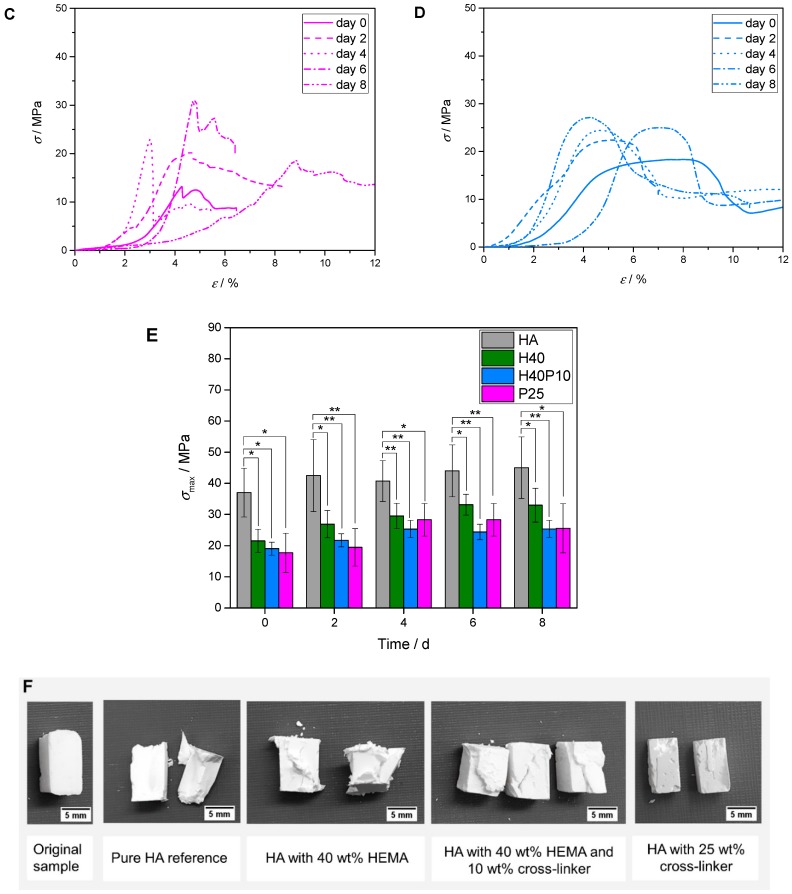
Stress-strain curves of inorganic reference in comparison to composite materials as well as overview of maximum stress-values over 8 d. (**A**) Inorganic reference (HA) with typical brittle behavior—high initial stress with low strain-levels; (**B**) Formulation containing 40 wt% HEMA in liquid phase (H40) showing an increase in ductility with high stress-values comparable to pure HA cement; (**C**) Samples based on 25 wt% cross-linker (P25) with increase in ductility; (**D**) Most ductile system composed of HEMA (40 wt%) and hydrogel precursor (10 wt%) with plateau phases instead of maximum stress peaks and deformations up to 10%; (**E**) Maximum stress of all batches during time frame of 8 d. Image (**F**) depicts specimens before and after compressive strength testing at day 0. * *p* < 0.05; ** *p* < 0.001.

Stress-strain curves for the inorganic reference without any polymeric content (HA) revealed the typical brittle behavior with a high stress of 40–50 MPa until immediate break and a corresponding strain maximum of about 2% (see Figure 11A). Incorporation of a pHEMA network in HA-matrix (H40) also resulted in a doubling of strain-values up to 4%. During eight days, the final conversion to HA and thus a higher entanglement of HA-needles resulted in higher stress-levels that were almost in the same range (~30–40 MPa) and thus comparable to pure HA, but more elastic (see Figure 11B). Hydrogels based on high molecular weight PEG-PLLA precursor with the softer gel phase led to even more ductile properties but at the same time lower maximum stress levels (see Figure 11C). By combining both stiffer pHEMA and softer PEG-PLLA-based hydrogels, an increase in elasticity with high energy up-take and a deformation up to 10% was achieved. With 40 wt% HEMA and 10 wt% cross-linker (H40P10), maximum stress points were converted to broad peaks or plateau phases (see Figure 11D). While having higher maximum compressive strength values for pure HA, all composite formulations showed still stable levels of about 50–75% compared to pure inorganic matrix. Over time, an increase was noted due to the further entanglement of HA crystal network within the hydrogel matrix (see Figure 11E). Additionally, a certain cohesiveness could be observed for composite samples after testing as the specimens did not disintegrate along the crack after mechanical testing (see Figure 11F).

## 4. Discussion

The polymerization of HEMA as a monofunctional methacrylate monomer results in the formation of long polymer chains, which form a hydrogel by the physical entanglement of the chains without further chemical cross-linking. The effect of HEMA modification on the mechanical properties of an apatite calcium phosphate cement was demonstrated in a previous study [12]. Here, we hypothesized that a further chemical cross-linking of HEMA by a bifunctional methacrylate monomer will further enhance the elasticity of the cement composites and will also enable hydraulic cleavage of the network by ester hydrolysis of the lactide moieties within the chemical structure.

By combining or replacing pHEMA with a PEG-PLLA-based hydrogel precursor, setting time was decreased significantly in comparison to pure HA. Both batches, H40P10 and P25, that were produced with the synthesized monomer, resulted in an even faster setting that only took halve of the time than the formulation with 40 wt% HEMA (H40) alone. This result correlates well with the determined gelation points of the pure hydrogel systems. Due to higher molecular weight of the monomers as well as the included bifunctionality, the cross-linker reacts and cross-links even faster via radical polymerization, even if the amount of single short chain HEMA monomers might be higher. For the bifunctional precursor, the extend of effective cross-links is increased and network formation is accelerated. Moreover, this logically also implements the HPLC study where an integration of over 90% of methacrylic acid units of hydrolyzed hydrogels was detected to be involved in gel structure. Consequently, the reaction of the high molecular weight precursor can be concluded as fast with a high reaction rate. This high degree of conversion in the polymerization was also investigated by Clapper et al. [17] who used an ultra-violet induced gelation of dimethacrylated triblock monomers of PEG and PLA. All these facts successfully demonstrated that the second gel forming reaction could run in parallel to the setting reaction without any restrictions. Also, the adhesiveness of the samples after compressive strength testing indicated a certain cohesiveness by incorporated hydrogel networks and inter-locked HA crystals. Nevertheless, the fast organic reaction did not inhibit or change the conversion of the inorganic part completely. We observed an influence on conversion rate and crystal size, but this did not affect mechanical stability or lead to a disintegration of the cement samples. XRD-diffractograms showed a continuous decrease in α-TCP pattern over the time with a successful setting reaction to HA. The detected increase in amorphousness with higher polymeric content as well as reduced transformation were also observed by Hurle et al. [18], who assumed a lower HA formation by a reduced availability of water. This results in less dissolution of α-TCP raw powder and consequently production of precipitated setting product.

Regarding mechanical properties, we could clearly increase toughness and elasticity by addition of the synthesized PEG-PLLA–based cross-linker as a third component in the system. A switch from brittle to ductile behavior was achieved and also bending strength increased in contrast to pure HA. Stress-strain curves of compressive strength testing also showed a high deformation for the batch H40P10. This also correlates with the homogenous testing sample that stuck together after compression. We could create a certain plateau that means a change in direction of the x-axis (strain) without altering the detected response or force on the y-axis (alternatively: stress). Thus, a high energy could be retained in the constructs. This was also seen for four-point bending testing and high toughness levels that were obtained for this formulation, which were comparable to the mechanical performance of pure HEMA cement composites from a study by Christel et al. [12] as well as acrylamide modified cements from the pioneering work of Dos Santos et al. [9]. With the addition of the degradable cross-linker, strength values were found to be in the range of samples containing 50 wt% HEMA but with the advantage of a degradable monomer component. This is in contrast to a study using the cross-linker in combination with more degradable brushite cement, where strength values decreased extremely compared to the addition of about 50 wt% PEG-hydrogel phase [13]. The high flexibility and deformation of such brushite composites resulted in values of less than 1 MPa for tensile strength, which can be correlated to the different shape, size and entanglement of cement crystals compared to apatite forming counterparts. The same trend was observed for brushite cement and silica gel hybrid materials [19] which were prepared via simultaneous hardening and gelation mechanism also showed lower levels in comparison to the here established formulations. It is hence obvious that the nanosized character of the precipitated hydroxyapatite crystals from the current study is responsible for the higher strength of cement – hydrogel composites. This is likely caused by a better entanglement of the smaller crystals within the polymeric network since for both cement types no chemical interaction between cement crystals and the hydrogel is expected.

An advantage of a high molecular weight PEG-PLLA-DMA–based cross-linker is the possibility of composite degradation in an aqueous environment. With additional ester linkages in the molecule, a hydrolytic degradation is enabled compared to the practically non-degradable HEMA matrix. At the same time, the ceramic’s resorption can occur due to an acidic environment with osteoclasts attaching at the surface and forming lacunas with subsequent phagocytosis and degradation of crystals by macrophages [20]. With this new approach, the resorption rate is increased over the time by an appropriate concentration of PEG-PLLA-DMA as seen for the mass change of the pure hydrogels. Cleavage of ester bonds occurs homogenously [21] within several weeks by bulk erosion [22] and can accelerate the overall degradation behavior of the composite.

## 5. Conclusions

Overall, we advanced a dual setting system based on HA and pHEMA formed via radical polymerization by incorporation of an additional degradable cross-linker. Ductile as well as elastic properties could be increased by only 10 wt% addition to a system based on HA and pHEMA (here: 40 wt% HEMA; H40P10). Pastes were still moldable though a relatively high polymeric content was embedded in the liquid phase. Moreover, hydrogel formation did not affect setting reaction, and both reactions were able to process. Further investigations are needed to optimize the final concentrations of both organic compounds. In conclusion, we could successfully create a promising tool for biomedical applications by incorporation of a degradable hydrogel precursor to improve the mechanical properties of a dual setting cement system.

## Figures and Tables

**Table 1:** Composition of the different liquid phases for composite formulations. H40 refers to 40 wt% HEMA monomer in the solution, whereas P10 corresponds to 10 wt% cross-linker addition. The combination of 40 wt% HEMA and 10 wt% cross-linker is abbreviated with H40P10.

Substances	Percentage by Weight/%		
Abbreviations	H40	P10	H40P10
HEMA	40	–	40
Cross-linker	–	10	10
2.5 % Na_2_HPO_4_-solution	59.25	89.25	49.25
APS	0.5		
TEMED	0.25		

APS: ammonium persulfate; HEMA: 2-hydroxyethyl methacrylate; TEMED: tetramethylethylenediamine.

## References

[1] Hing K.A. (2004). Bone repair in the twenty–first century: Biology, chemistry or engineering?. Philos. Trans. R. Soc. Lond. A Math. Phys. Eng. Sci..

[2] Olszta M.J., Cheng X., Jee S.S., Kumar R., Kim Y.Y., Kaufman M.J., Douglas E.P., Gower L.B. (2007). Bone structure and formation: A new perspective. Mater. Sci. Eng. R Rep..

[3] Weiner S., Wagner H.D. (1998). The material bone: Structure-mechanical function relations. Annu. Rev. Mater. Sci..

[4] Barinov S., Komlev V. (2011). Calcium phosphate bone cements. Inorg. Mater..

[5] Ginebra M.-P., Espanol M., Montufar E.B., Perez R.A., Mestres G. (2010). New processing approaches in calcium phosphate cements and their applications in regenerative medicine. Acta Biomater..

[6] Ramesh N., Moratti S.C., Dias G.J. (2018). Hydroxyapatite–polymer biocomposites for bone regeneration: A review of current trends. J. Biomed. Mater. Res. Part B Appl. Biomater..

[7] Bohner M. (2000). Calcium orthophosphates in medicine: From ceramics to calcium phosphate cements. Injury.

[8] Geffers M., Groll J., Gbureck U. (2015). Reinforcement Strategies for Load-Bearing Calcium Phosphate Biocements. Materials.

[9] Dos Santos L.A., Carrodeguas R.G., Boschi A.O., de Arruda A.C. (2003). Dual-Setting Calcium Phosphate Cement Modified with Ammonium Polyacrylate. Artif. Organs.

[10] Wang J., Liu C., Liu Y., Zhang S. (2010). Double-Network Interpenetrating Bone Cement via in situ Hybridization Protocol. Adv. Funct. Mater..

[11] Schamel M., Groll J., Gbureck U. (2017). Simultaneous formation and mineralization of star-P (EO-stat-PO) hydrogels. Mater. Sci. Eng. C Mater. Biol. Appl..

[12] Christel T., Kuhlmann M., Vorndran E., Groll J., Gbureck U. (2013). Dual setting α-tricalcium phosphate cements. J. Mater. Sci. Mater. Med..

[13] Rödel M., Teßmar J., Groll J., Gbureck U. (2018). Highly flexible and degradable dual setting systems based on PEG-hydrogels and brushite cement. Acta Biomater..

[14] Sawhney A.S., Pathak C.P., Hubbell J.A. (1993). Bioerodible hydrogels based on photopolymerized poly (ethylene glycol)-co-poly (α-hydroxy acid) diacrylate macromers. Macromolecules.

[15] Marie A., Fournier F., Tabet J. (2000). Characterization of synthetic polymers by MALDI-TOF/MS: Investigation into new methods of sample target preparation and consequence on mass spectrum finger print. Anal. Chem..

[16] Abbadessa A., Mouser V.H.M., Blokzijl M.M., Gawlitta D., Dhert W.J.A., Hennink W.E., Malda J., Vermonden T. (2016). A synthetic thermo-sensitive hydrogel for cartilage bioprinting and its biofunctionalization with polysaccharides. Biomacromolecules.

[17] Clapper J.D., Skeie J.M., Mullins R.F., Guymon C.A. (2007). Development and characterization of photopolymerizable biodegradable materials from PEG–PLA–PEG block macromonomers. Polymer.

[18] Hurle K., Christel T., Gbureck U., Moseke C., Neubauer J., Goetz-Neunhoeffer F. (2016). Reaction kinetics of dual setting α-tricalcium phosphate cements. J. Mater. Sci. Mater. Med..

[19] Geffers M., Barralet J.E., Groll J., Gbureck U. (2015). Dual-setting brushite–silica gel cements. Acta Biomater..

[20] Wenisch S., Stahl J.P., Horas U., Heiss C., Kili O., Trinkaus K., Hild A., Schnettler R. (2003). In vivo mechanisms of hydroxyapatite ceramic degradation by osteoclasts: Fine structural microscopy. J. Biomed. Mater. Res. Part A.

[21] Browning M.B., Cereceres S.N., Luong P.T., Cosgriff-Hernandez E.M. (2014). Determination of the in vivo degradation mechanism of PEGDA hydrogels. J. Biomed. Mater. Res. Part A.

[22] Metters A.T., Bowman C.N., Anseth K.S. (2000). A statistical kinetic model for the bulk degradation of PLA-b-PEG-b-PLA hydrogel networks. J. Phys. Chem. B.

